# Acute Afterload Stress Induced by Phenylephrine Administration Reveals Ventricular Dysfunction in Myocardial Infarcted Female Rats Previously Exposed to Cigarette Smoke

**DOI:** 10.3390/biomedicines14102328

**Published:** 2026-10-13

**Authors:** Danilo S. Bocalini, Andre S. Leopoldo, Ana Paula Lima-Leopoldo, Bárbara do Vale, Maria Eduarda de Almeida, Manolo D. dos Anjos, Alexandre César Fioretti, Patrik Aronsson, Monica A. Sato

**Affiliations:** 1 Department of Morphology and Physiology, Faculdade de Medicina do ABC, Centro Universitário FMABC, Santo Andre 09060-650, SP, Brazil; bocaliniht@hotmail.com (D.S.B.); monica.sato@fmabc.net (M.A.S.); 2 Experimental Biochemistry and Physiology Laboratory, Federal University of Espírito Santo, Vitoria 29075-910, ES, Brazil; 3 Department of Pharmacology, Institute of Neuroscience and Physiology, University of Gothenburg, Sahlgrenska Academy, SE-405 30 Gothenburg, Sweden

**Keywords:** myocardial infarction, cigarrette, ventricular function, hemodynamic, cardiac assessment

## Abstract

**Background/Objective:** This study investigated the hemodynamic responses to acute pressure overload in female rats with myocardial infarction (MI) previously exposed to cigarette smoke (CS). **Methods:** Female Wistar rats were allocated into four groups: sham (S, *n* = 10), Smoker (SS, *n* = 8), myocardial infarction (MI, *n* = 9), and smoker with myocardial infarction (SMI, *n* = 10). The S and MI groups were housed under standard conditions for 16 weeks, whereas the SS and SMI groups were exposed to CS for 16 weeks. Subsequently, MI was induced by coronary artery occlusion (CAO), while sham-operated animals underwent the corresponding surgical procedure without coronary occlusion. Left ventricular pressure was measured using a micromanometer introduced through right common carotid artery catheterization, with the transducer positioned within the left ventricular cavity. Ascending aortic flow was simultaneously recorded. Hemodynamic signals were acquired using the AcqKnowledge^®^ system to determine left ventricular systolic pressure (LVSP), left ventricular end-diastolic pressure (LVEDP), heart rate, first derivative positive (+dP/dt) and negative (−dP/dt) of left ventricular pressure, cardiac output, stroke volume (SV), and systolic work (SW). Systolic work was calculated as SW = (LVSP − LVEDP) × SV × 0.0136. **Results:** At baseline, significant differences were observed in dP/dt_max, with the highest values in SS rats (10610.00 ± 1160.00 mmHg/s), followed by S (8663.00 ± 767.50 mmHg/s), MI (6054.00 ± 1122.10 mmHg/s), and SMI rats (4611.00 ± 1100.50 mmHg/s). Similarly, stroke work index was reduced in infarcted animals, with the lowest values observed in the SMI (0.46 ± 0.3 gm·min^−1^/kg) compared to other groups (S:0.85 ± 0.17, SS: 0.84 ± 0.17, MI: 0.65 ± 0.14; gm·min^−1^/kg). Following acute pressure overload induced by phenylephrine, indexed stroke work index differed significantly among groups (S: 71.72 ± 6.49%; SS: 28.05 ± 25.02%; MI: −16.00 ± 29.47%; SMI: −50.00 ± 16.22%; *p* < 0.0001). These findings demonstrate that ventricular functional reserve is markedly impaired in rats with MI previously exposed to CS. **Conclusions:** Prior exposure to cigarette smoke appears to exacerbate the cardiac dysfunction associated with myocardial infarction, particularly under conditions of acute increases in ventricular afterload.

## 1. Introduction

Cigarette smoking (CS) remains a major modifiable determinant of cardiovascular morbidity and mortality and is strongly associated with myocardial infarction and subsequent heart failure. Its cardiovascular toxicity extends beyond atherosclerotic plaque formation and thrombosis: cigarette smoke promotes endothelial dysfunction, oxidative stress, inflammation, autonomic disturbance, and adverse myocardial remodeling, thereby affecting both vascular loading conditions and intrinsic cardiac performance [1,2,3]. The clinical relevance of exposure history is substantial because the risk associated with tobacco use decreases after cessation but does not necessarily return immediately to that of individuals who have never smoked [4].

Myocardial infarction (MI) initiates a complex remodeling process involving infarct expansion, chamber dilation, compensatory hypertrophy, altered ventricular compliance, and progressive systolic and diastolic dysfunction. These adaptations may initially preserve output but can ultimately increase filling pressures, promote pulmonary congestion, and reduce cardiac reserve [5]. Resting measurements are therefore not always sufficient to characterize the functional consequences of myocardial injury, particularly when compensatory mechanisms maintain apparently acceptable pressure or flow under basal conditions.

Experimental studies indicate that (CS) exposure can induce cardiac hypertrophy, fibrosis, oxidative and inflammatory signaling, and impaired myocardial mechanics [6,7,8,9,10,11,12,13,14,15]. This issue is especially relevant when a subsequent myocardial infarction occurs after a period of chronic smoke exposure, because pre-existing structural, vascular, or cellular alterations could reduce the heart’s capacity to adapt to ischemic injury even in the absence of continued exposure.

Cardiac functional reserve can be experimentally assessed by imposing a controlled acute increase in ventricular afterload. In rats with post-infarction heart failure, phenylephrine-induced pressure overload following baroreflex interruption has been used to unmask ventricular dysfunction that may not be fully apparent under basal conditions [16]. Accordingly, the combined assessment of basal hemodynamics and the ventricular response to a standardized afterload challenge enables the distinction between established resting dysfunction and impaired functional reserve, reflected by an inadequate ability to augment myocardial performance and maintain effective forward flow in response to increased ventricular loading.

Despite the recognized cardiovascular consequences of smoking, limited evidence addresses whether prior cigarette-smoke exposure leaves residual hemodynamic vulnerability after myocardial infarction in female animals. In this perspective, studies evaluating the consequences of cigarette smoke exposure prior to MI are scarce in experimental models [17,18]. Accordingly, this study investigated the structural, echocardiographic, and invasive hemodynamic repercussions of previous (CS) exposure in female rats with post-MI, both at baseline and during an acute phenylephrine-induced increase in afterload.

## 2. Materials and Methods

### 2.1. Animals

A total of 37 female (3 months of age) Wistar rats (*Rattus norvegicus albinus*, Rodentia, Mammalia) were obtained from the animal facility and experimental laboratory of ABC Medical School University Center, Brazil, and were randomly allocated into four experimental groups. Sham (S; *n* = 10): animals maintained under standard housing conditions for 16 weeks, subsequently subjected to sham surgery without coronary artery occlusion, and then allowed to recover for an additional 8 weeks. Smoker (SS; *n* = 8): animals exposed to cigarette smoke for 16 weeks, subjected to sham surgery without coronary artery occlusion, and subsequently maintained for an additional 8 weeks without cigarette smoke exposure. Myocardial infarcted (MI; *n* = 9): animals maintained under standard housing conditions for 16 weeks and subsequently subjected to permanent coronary artery occlusion to induce myocardial infarction, followed by 8 weeks of recovery. Smoker and myocardial infarcted (SMI; *n* = 10): animals exposed to cigarette smoke for 16 weeks and subsequently subjected to permanent coronary artery occlusion. All animals were housed in plastic cages under controlled temperature and light–dark cycle conditions, with free access (ad libitum) to water and standard chow pellets (Nuvital^®^, Nuvilab, Brazil). A maximum of five animals were housed per cage. Thereafter, animals remained in recovery for 8 weeks without further cigarette smoke exposure. The study was reported in accordance with the ARRIVE guidelines [19] for animal research, and all experimental procedures were conducted in accordance with the applicable institutional and national regulations governing the care and use of laboratory animals. All procedures performed were in accordance with the Guide for the Care and Use of Laboratory Animals (NIH) and the ethical standards of the Experimentation and Ethics Committee on Animal Use of the Federal University of Sao Paulo (protocol code 0340/08; approved in 16 May 2008).

### 2.2. Cigarette Smoke Exposure

Rats were exposed to cigarette smoke in a chamber (dimensions: 1:000 × 800 × 700 mm) connected to a smoking device according to other studies [6,20]. The smoke was drawn out of filtered commercial cigarettes (composition per unit: 1.1 mg nicotine, 14 mg tar, and 15 mg carbon monoxide) with a vacuum pump and exhausted into the smoking chamber. In the first week, the number of cigarettes was gradually increased from 5 to 20 cigarettes for 60 min, administered twice a day. Subsequently, 20 cigarettes were used in each smoking session, twice a day (60 min in the morning and 60 min in the afternoon) for 16 weeks. The chamber carbon monoxide content was measured with a sensor Toxi Vision CO 860 (Biosystems, Prairieville, LA, USA).

### 2.3. Myocardial Infarction (MI) Protocol

Experimental MI was produced according to a standardized model utilized by our group [21]. Briefly, animals were anesthetized with a mixture of ketamine (50 mg/kg i.p.) and xylazine (10 mg/kg i.p.), and after orotracheal intubation (Gelko-14G, Gelko, Incheon, Republic of Korea), mechanical ventilation was instituted with a ventilator (Rodent ventilator Mod 683, Harvard Apparatus©, Boston, MA, USA) with a frequency of 90 movements/minute and a tidal volume of 2.0 mL. Through a left thoracotomy using the third intercostal space as a reference, the heart was exteriorized and the anterior interventricular branch of the left coronary artery was occluded at the level of the lower border of the left atrium with 6.0 mononylon thread, causing permanent arterial occlusion. Next, the heart was internalized and the thorax was immediately closed using a purse-string ligature, previously prepared. Thereafter, animals were removed from artificial ventilation and natural breathing was stimulated.

### 2.4. Evaluated Parameters

#### 2.4.1. Echocardiographic Assessment

Transthoracic echocardiography was performed in all animals under the same anesthetic protocol used for coronary occlusion surgery (mixture of ketamine 50 mg/kg i.p. and xylazine 10 mg/kg i.p.) and following thoracic shaving, according to laboratory-standardized procedures [20,21]. After 8 weeks of surgery, the surviving animals supposedly infarcted were submitted to echocardiography to confirm MI by detecting and measuring the extent of MI in relation to the LV wall. Examination was done by one observer blinded to the animal group, with an HP SONOS 5500 (Philips Medical Systems, Andover, MA, USA) and a 12 MHz/2 cm transducer, according to a previously detailed technique [16,22]. The infarct size was measured by echocardiography at basal, mid, and apical LV levels, by analyzing the length of the arc corresponding to the hypokinetic and/or akinetic portion (infarcted area) and the total perimeter of the LV endocardial border at the end of diastole. Thus, the relative MI size was expressed in % of the LV averaged in three planes. The MI size was distributed into small (0 to 29%), moderate (30 to 39%) and large (>40%) of LV. However, according to previous findings [23], female rats with myocardial infarction involving less than 37% of the left ventricular (LV) perimeter do not exhibit marked pathological consequences. For this reason, in this study, animals with a scar arc occupying more and less than 35% of the LV perimeter were also evaluated.

#### 2.4.2. Hemodynamic and Acute Afterload Stress Study

After 24 h of transthoracic echocardiography evaluation, the hemodynamic evaluation was performed on a heated operating table (37 °C), under adjusted anesthesia (urethane chloride, 1.5 g/kg i.v., Sigma-Aldrich, St. Louis, MO, USA) and oxygen-enriched ventilation with a closed chest according to a previous publication [16]. The left femoral vein was accessed for drug or saline administration. A micromanometer (MikroTip 2F; Millar Instruments Inc., Houston, TX, USA) was inserted from the right carotid artery into the LV cavity to assess the intraventricular pressure, and a flow ultrasound probe (Transonic Systems Inc., Ithaca, NY, USA) was positioned around the ascending aorta, after a brief right thoracotomy, to estimate the LV ejection. The following data were acquired and analyzed (Acknowledge Software, Biopac Systems, Santa Barbara, Calif.): LV systolic (SP) and end-diastolic pressures (EDPs), rate of change in LV pressure (dP/dt), heart rate (HR), cardiac output (CO), and stroke volume (SV). Stroke work (SW) was estimated as the product of SV × (SP − EDP) × 0.0136.

In accordance with the protocol described by dos Santos et al. [16], after baseline assessment, baroreflex blockade was induced by bilateral vagotomy, followed by an acute afterload challenge achieved through pharmacologically induced pressure overload with phenylephrine (PHE; L-phenylephrine hydrochloride, Sigma-Aldrich). The PHE dose (15–25 µg/kg, i.v.) was individually adjusted to achieve a comparable increase in arterial blood pressure of 50–70% above baseline.

#### 2.4.3. Pulmonary Congestion

At the end of the experiments, the animals were euthanised by deep anesthesia with an overdose of 2.0 g/kg iv urethane (Sigma-Aldrich, St. Louis, MO, USA) according to Ogihara et al. [23]. After euthanasia, the right lung was isolated by placing a cotton ligature around the pulmonary hilum to prevent fluid loss during tissue handling. The lung was immediately excised and weighed to obtain the wet mass. Subsequently, the tissue was dried in an oven at 85 °C for 12 h and reweighed to determine the dry mass. Lung water content was calculated using the following equation: lung water content (%)= [(wet mass − dry mass)/wet mass] x 100. The lung water content was determined as a sensitive indicator of pulmonary congestion, according to previously described methods [16].

### 2.5. Statistical Analyses

Data distribution and normality were assessed using the D’Agostino–Pearson omnibus normality test. Comparisons between two independent groups were performed using an unpaired Student’s *t*-test. For analyses involving two independent factors, two-way analysis of variance (ANOVA) was performed, followed by Bonferroni’s post hoc test when appropriate. For non-parametric data involving more than two groups, the Kruskal–Wallis test was applied, followed by Dunn’s multiple-comparison post hoc test when appropriate. Continuous data are expressed as mean ± standard error of the mean (SEM). All statistical analyses were performed using GraphPad Prism version 4.0 (GraphPad Software, Inc., San Diego, CA, USA), and statistical significance was set at *p <* 0.05.

## 3. Results

Similar values were observed for body mass (S: 249.00 ± 13.86 g, SS: 241.80 ± 16.78 g, MI: 253.90 ± 25.11 g, SMI: 265.70 ± 18.81 g; CS effect: *p* = 0.6441; MI effect: *p* = 0.0223; CS * MI: *p* = 0.1671). Echocardiographic data are presented in Table 1. Similar values were observed (*p* > 0.05) for MI size between MI and SMI. Heart rate did not differ among the experimental groups. No significant differences were observed in the diastolic or systolic area between the S and SS groups. However, both infarcted groups exhibited significantly higher values than the MI and SMI groups, which also differed significantly from each other.

Diastolic filling was assessed using peak E-wave and A-wave velocities and the transmitral E/A ratio (Table 1). No significant differences were observed in E-wave velocity between the S, SS, and MI groups; however, the SMI group exhibited higher values compared with the S group. A-wave velocity did not differ between the S and SS groups; nevertheless, these values were significantly higher than those observed in the MI and SMI groups, which did not differ from one another. Similarly, the E/A ratio differed significantly among groups. The MI and SMI groups exhibited significantly higher E/A ratio values compared with the S and SS groups, whereas the latter two groups showed similar values.

No differences were found between groups in body mass; however, after normalization of cardiac chamber weights to body weight, atrial weight was similar between the S and SS groups, and only the S group was significantly lower than the MI and SMI groups, which differed from each other. Right ventricular mass was comparable between the S, SS and MI groups and significantly lower than the SMI group. In contrast, both left ventricular mass and total cardiac mass of the S and SS groups differed from those of the MI and SMI groups, which differed from each other, indicating an exacerbation of cardiac hypertrophy in infarcted animals previously exposed to cigarette smoke. Pulmonary congestion, assessed by pulmonary water content, was significantly increased only in the MI and SMI groups.

The hemodynamic data obtained from the baseline assessment of the right and left ventricles are presented in Table 2. Regarding the effects on the right ventricle (RV), systolic pressure was significantly lower in the S and SS groups compared to in the MI and SMI groups, with the last two groups without significant difference from each other. Diastolic pressure was significantly higher in the MI and SMI groups than in the S group, whereas no significant differences were observed between the MI and SMI groups or between these groups and the SS group. The +dP/dt were similar between S and SS groups, and higher than MI and SMI that no differing significantly from each other. The −dP/dt were significantly higher in the S group in comparison with the SS, MI, and SMI groups, which did not differ significantly from one another.

No significant differences were observed in heart rate or left ventricular systolic pressure among groups (Table 2). However, left ventricular end-diastolic pressure was significantly higher in the MI and SMI groups compared to the S and SS groups, whereas no significant differences were observed between the MI and SMI groups or between the S and SS groups. The +dP/dt was significantly higher in the S group than in the SS, MI, and SMI groups, with all three groups differing significantly from one another. The −dP/dt was significantly higher in the S and SS groups than in the MI and SMI groups, with no significant differences within both pairs of groups. Cardiac output was significantly lower only in the SMI group compared with the S group, whereas the remaining groups did not differ significantly (Table 2). The cardiac index was significantly lower in the MI and SMI groups than in the S group, with no significant difference between the MI and SMI groups. No significant differences were observed in stroke volume. Nevertheless, the stroke volume index was significantly lower in the SMI group than in the S, SS, and MI groups, which did not differ significantly from one another. Systolic work was significantly lower in the SMI group than in the S and SS groups, but was similar to the MI group. Likewise, the systolic work index was significantly lower in the MI and SMI groups than in the S group, with no significant difference between the MI and SMI groups; however, only the SMI group exhibited a significantly lower value than the SS group.

**Figure 1:**
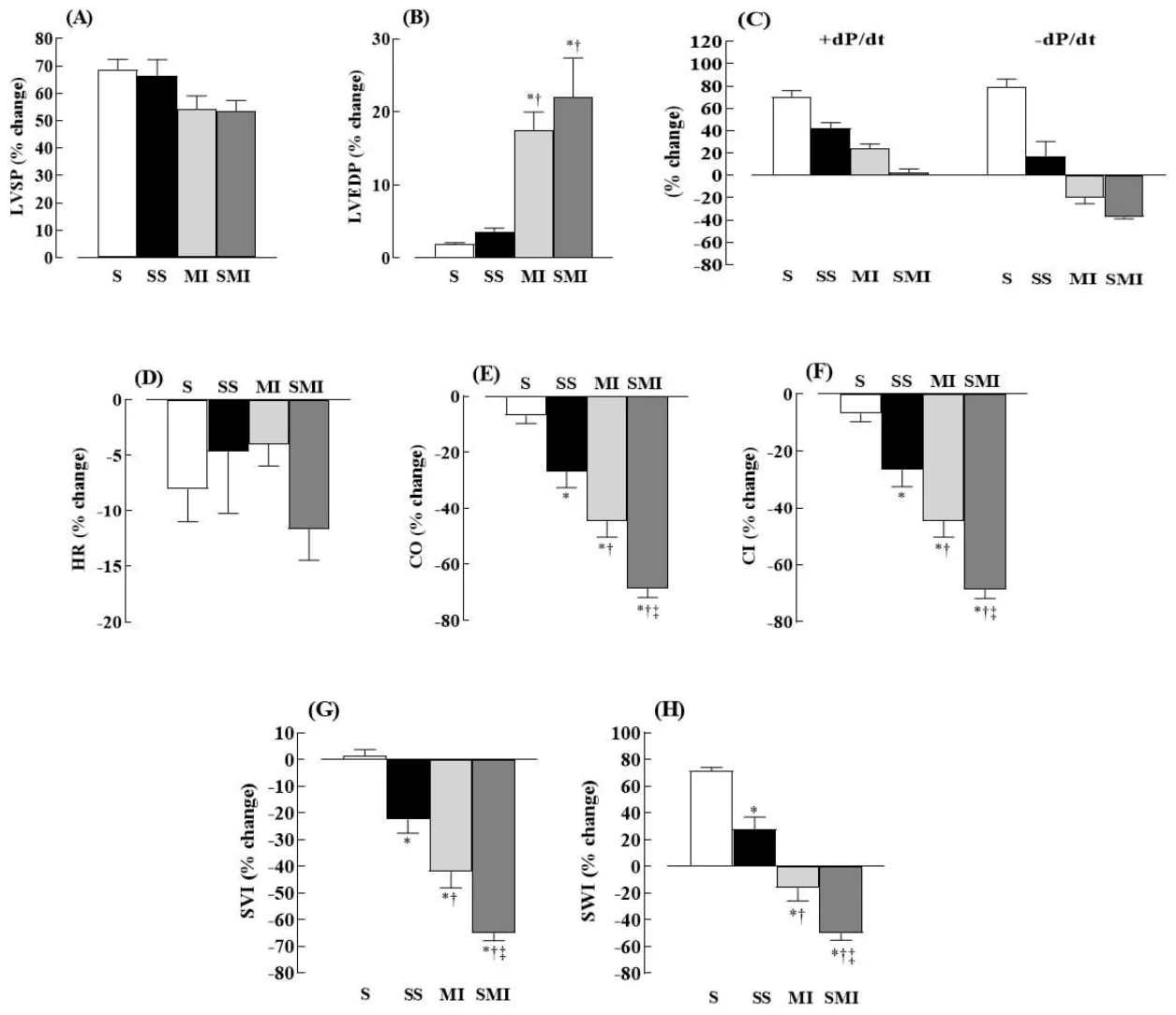
Values expressed as mean ± standard error of the relative effects after phenylephrine-induced increase in afterload in the sham (S), smoker (SS), myocardial infarcted (MI) and smoker + myocardial infarcted (SMI) groups. (**A**): left ventricular systolic pressure (LVSP). (**B**): left ventricular end-diastolic pressure (LVEDP). (**C**): first derivative positive (+dP/dt) and negative (−dP/dt) of left ventricular pressure. (**D**): heart rate (HR). (**E**): cardiac output (CO). (**F**): cardiac index (CI). (**G**): stroke volume index (SVI). (**H**): stroke work index (SWI). * vs. S group (*p* < 0.05) † vs. SS group (*p* < 0.05). ‡ vs. MI group (*p* < 0.05).

The increase in systemic arterial pressure induced by bolus phenylephrine administration elicited marked hemodynamic changes in all groups. However, the cardiac response to the acute elevation in arterial pressure was significantly attenuated in the S group compared with the SS, MI and SMI groups (Figure 1). Baroreflex blockade induced by vagotomy did not result in a significant reflex bradycardic response among the groups (S: −10.00 ± 3.62 bpm; SS: −12.89 ± 4.786 bpm; MI: −10.60 ± 4.78 bpm; SMI: −11.73 ± 1.67 bpm; CS effect: *p* = 0.1221, MI effect: *p* = 0.6134, CS * MI: *p* = 0.1976).

Phenylephrine was individually titrated to produce a broadly comparable increase in arterial pressure within a predefined target range of 50–70%. The increase in systolic arterial pressure evoked by phenylephrine was similar among all groups (S: 68.78 ± 8.58%, SS: 66.43 ± 16.68%, MI: 54.24 ± 13,63%, SMI: 53.53 ± 12.43%; CS effect: *p* = 0.7967, MI effect: *p* = 0.0118, CS * MI: *p* = 0.9657), indicating that the cardiac afterload imposed by phenylephrine administration was comparable across the experimental groups.

The MI and SMI groups exhibited a marked increase in relative values of LVEDP (MI: 15.56 ± 7.26%, SMI: 22.09 ± 16.72%) compared with the S (1.85 ± 0.69%) and SS (3.62 ± 1.40%) groups, which did not differ significantly from each other (CS effect: *p* = 0.5993, MI effect: *p* < 0.0001, CS * MI: *p* = 0.9897). No significant differences were observed in HR after phenylephrine in vagotomized rats (S: −8.01 ± 7.93%, SS: −4.65 ± 15.86%, MI: −3.97 ± 5.92%, SMI: 11.67 ± 8.82%; CS effect: *p* = 0.3545, MI effect: *p* = 0.4640, CS * MI: *p* = 0.1161). Significant differences were observed in the percentage increase in +dP/dt (S: 70.07 ± 15.70%; SS: 42.38 ± 14.13%; MI: 24.31 ± 11.80%; SMI: 2.31 ± 11.16%; CS effect: *p* < 0.0001, MI effect: *p* = <0.0001, CS * MI: *p* = 0.2992) and in the percentage change in −dP/dt (S: 79.43 ± 18.33%; SS: 16.81 ± 38.71%; MI: −19.54 ± 17.33%; SMI: −37.52 ± 4.36%; CS effect: *p* < 0.0001, MI effect: *p* < 0.0001, CS * MI: *p* = 0.0052) among the experimental groups.

Differences were found in cardiac output (CS effect: *p* < 0.0001, MI effect: *p* < 0.0001, CS * MI: *p* = 0.8466); thus, the values of SMI (−68.70 ± 9.86%) were higher than MI (−44.78 ± 16.81%), SS (−26.63 ± 16.58%) and S (−6.80 ± 7.51%). Significant differences were also detected in the cardiac index (S: −6.80 ± 7.51%; SS: −26.50 ± 16.61%; MI: −44.78 ± 16.81%; SMI: −68.70 ± 9.86%; CS effect: *p* < 0.0001, MI effect: *p* < 0.0001, CS * MI: *p* = 0.8356), stroke volume index (S: 1.63 ± 5.78%; SS: −22.42 ± 14.46%; MI: −42.11 ± 17.89, SMI: −65.00 ± 9.28%; CS effect: *p* < 0.0001, MI effect: *p* < 0.0001, CS * MI: *p* = 0.7222), and stroke work index (S: 71.72 ± 6.49%; SS: 28.05 ± 25.02%; MI: −16.00 ± 29.47%; SMI: −50.00 ± 16.22%; CS effect: *p* < 0.0001, MI effect: *p* < 0.0001, CS * MI: *p* = 0.3845), indicating progressive impairment of left ventricular function in the SS, MI, and SMI groups compared with the S group.

## 4. Discussion

Phenylephrine is a selective α1-adrenergic receptor agonist widely used to induce arterial vasoconstriction and increase systemic vascular resistance, thereby providing a controlled pharmacological approach for experimentally increasing ventricular afterload. Following intravenous administration, it produces a rapid and predictable pressor response, allowing precise and titratable manipulation of cardiovascular loading conditions [24]. Although its predominant effect is an increase in arterial vascular resistance and afterload, phenylephrine may also influence cardiac preload by promoting venous return, with this response depending on the prevailing preload status and position on the Frank–Starling relationship [25,26]. Its rapid and reproducible hemodynamic effects make phenylephrine particularly suitable for experimental protocols designed to investigate ventricular functional reserve under acute afterload stress. Pharmacokinetically, phenylephrine undergoes extensive metabolism, primarily through monoamine oxidase, and, because it lacks a catechol ring, is not substantially metabolized by catechol-O-methyltransferase, contributing to its relatively stable pharmacological profile [24]. Collectively, these pharmacological properties make phenylephrine a suitable agent for inducing controlled and transient increases in arterial pressure and ventricular afterload in cardiovascular research.

Beyond its well-established vasoconstrictor effects, phenylephrine has become an important experimental tool for investigating cardiac functional reserve [16]. Acute α1-adrenergic receptor stimulation increases systemic vascular resistance and left ventricular afterload, thereby requiring the myocardium to generate higher systolic pressure in order to preserve stroke volume and cardiac output. In healthy hearts, this physiological challenge is effectively compensated by enhanced contractility, allowing maintenance of global cardiac performance. Conversely, hearts undergoing pathological remodeling or developing heart failure exhibit limited contractile reserve, resulting in an impaired ability to adapt to acute increases in afterload [16]. Consequently, phenylephrine-induced afterload stress provides a more sensitive assessment of ventricular performance than resting hemodynamic measurements alone, revealing subtle impairments in pressure-generating capacity (+dP/dt), cardiac output, stroke volume, and myocardial work that may remain concealed under basal conditions. This approach is particularly valuable in experimental models of heart failure, where compensatory neurohumoral and structural mechanisms frequently preserve resting cardiovascular function despite substantial deterioration of intrinsic myocardial performance. Therefore, phenylephrine-induced hemodynamic stress has emerged as a robust and physiologically relevant strategy for evaluating ventricular functional reserve and myocardial adaptation to increased mechanical load [16,24].

In the present study, acute phenylephrine-induced hemodynamic overload proved to be a highly sensitive experimental approach for identifying functional abnormalities that were only partially detectable during baseline evaluation. Although resting hemodynamic parameters provide valuable information regarding cardiovascular status, they do not necessarily reflect the myocardium’s capacity to respond to acute increases in ventricular afterload, which represents a fundamental component of cardiac functional reserve [16]. Importantly, all experimental groups exposed either to cigarette smoke, myocardial infarction, or the combined intervention exhibited a diminished adaptive response to pharmacological stress compared with controls, despite displaying less pronounced alterations under resting conditions. This finding highlights the limitations of conventional basal hemodynamic assessment and underscores the importance of functional stress testing for uncovering latent myocardial dysfunction.

The principal finding of this study indicates that previous cigarette-smoke exposure was associated with a more adverse post-infarction phenotype in female rats, particularly when cardiac performance was challenged by an acute increase in afterload. Infarct size was comparable between the MI and SMI groups, yet SMI animals exhibited greater atrial, left ventricular, and total cardiac mass, larger left ventricular cavity areas, a higher E/A ratio, lower basal indexes of forward performance, and the most pronounced reductions in cardiac output, cardiac index, stroke-volume index, and stroke-work index during phenylephrine stress. By contrast, the SS group showed relatively preserved resting chamber dimensions and global flow, but already displayed abnormalities in selected contractile–relaxation indexes and an attenuated response to afterload. Together, these findings support the interpretation that prior smoke exposure may leave a residual cardiovascular substrate that is incompletely expressed at rest, but becomes evident when the post-infarcted heart is required to increase performance.

The structural findings are consistent with advanced post-infarction remodeling. Myocardial infarction characteristically produces chamber dilation, altered geometry, and compensatory hypertrophy, which may progress from adaptive compensation to heart failure [5,26]. Both infarcted groups showed enlarged systolic and diastolic areas, reduced fractional area shortening, increased cardiac mass, and pulmonary water accumulation, confirming a substantial heart failure phenotype. The larger atrial, left ventricular, and total cardiac masses in SMI compared with MI, despite similar infarct size, suggest that differences in scar extent alone are unlikely to explain the more pronounced remodeling. However, the present data do not establish the cellular composition of the increased mass; cardiomyocyte hypertrophy, fibrosis, edema, or their combination would require histological or molecular confirmation.

The atrial enlargement observed in the infarcted groups is compatible with chronically elevated left ventricular filling pressure and volume stress. The higher atrial mass in the SMI group may indicate a greater cumulative filling-pressure burden or reduced ventricular compliance, whereas the increase in right ventricular mass and systolic pressure is consistent with secondary pressure loading arising from left-sided failure and pulmonary congestion. Because pulmonary vascular resistance and lung histology were not measured, the right ventricular findings should not be attributed to a specific pulmonary vascular mechanism. Nonetheless, the combination of increased lung water, higher right ventricular systolic pressure, and impaired right ventricular pressure development supports the presence of biventricular consequences of the post-infarction syndrome.

The echocardiographic profile indicates marked systolic dysfunction in the MI and SMI groups and alterations in ventricular filling, particularly in the SMI group. The reduced fractional area shortening in both infarcted groups is consistent with the loss of contractile myocardium and chamber dilation. The increased E/A ratio in the SMI group, accompanied by reduced A-wave velocity and elevated invasive left ventricular end-diastolic pressure, is compatible with altered ventricular filling and increased filling pressure. However, the transmitral E/A ratio alone does not permit definitive discrimination among impaired relaxation, pseudonormalization, and restrictive filling patterns, particularly in anesthetized rats with high heart rates. Therefore, these findings should be interpreted as evidence of altered ventricular filling rather than as definitive evidence of a specific form of diastolic dysfunction. Tissue Doppler indices, E-wave deceleration time, left-atrial dimensions, and additional invasive or serial assessments would provide a more comprehensive characterization of diastolic function. The absence of these complementary measurements represents an important limitation of the present study. The echocardiographic assessment of ventricular function should ideally integrate complementary systolic and diastolic parameters, as isolated indices may not fully characterize ventricular performance [27]. In the present study, tissue Doppler, deceleration time, left-atrial dimensions, and serial assessments were not available, which limits a more comprehensive characterization of diastolic function.

At baseline, the combined-exposure group showed the clearest impairment in forward flow: cardiac output was lower than in sham rats, while stroke-volume index and stroke work were reduced relative to several comparison groups. The lower left ventricular +dP/dt in SMI than MI groups suggests more severe impairment of pressure-generating capacity, whereas the reduction in −dP/dt in infarcted animals is compatible with impaired relaxation. The smoke-only group is noteworthy, as resting cardiac output and stroke volume remained preserved despite alterations in pressure-derivative responses. This dissociation is plausible in compensated remodeling, in which heart rate, preload, vascular tone, and chamber geometry temporarily maintain global output despite impaired intrinsic myocardial mechanics. Experimental smoke studies have similarly reported hypertrophic remodeling, oxidative/inflammatory activation, fibrosis, and impaired myocardial mechanics [6,7,8]. These pathways are biologically plausible contributors here, but they remain hypotheses because oxidative stress, inflammation, fibrosis, endothelial function, and autonomic activity were not directly measured. However, the important to mentioned that endothelial dysfunction may also represent an important contributor to the altered cardiovascular response observed in this model, given its established association with adverse cardiovascular remodeling and prognosis [28,29]. However, because endothelial function was not directly assessed in the present study, this mechanism should be regarded as a plausible hypothesis rather than a demonstrated pathway.

The afterload challenge provides the strongest evidence of impaired ventricular functional reserve under acute hemodynamic stress. Phenylephrine administration primarily increases systemic vascular resistance and arterial pressure, thereby imposing an acute increase in ventricular afterload; however, changes in venous return and preload may also contribute to the resulting hemodynamic response. Therefore, changes in +dP/dt, cardiac output, stroke volume, and stroke work should not be interpreted as isolated measures of intrinsic myocardial contractility. Rather, their integrated response reflects the ability of the cardiovascular system to adapt to an acute increase in ventricular loading. Because the relative increase in systolic pressure following phenylephrine administration was broadly comparable among groups, the progressive deterioration in output- and work-related responses from S to MI and SMI indicates a markedly impaired ventricular functional response to afterload stress. Under this challenge, control animals increased +dP/dt and stroke-work index while largely preserving cardiac output, whereas smoke-exposed and infarcted animals exhibited progressively greater reductions in cardiac output, stroke-volume index, and stroke-work index. Thus, the present findings indicate a reduced capacity to maintain effective forward flow and ventricular performance when exposed to an acute increase in afterload, rather than providing direct evidence of impaired intrinsic myocardial contractile reserve. Definitive assessment of load-independent contractility would require complementary approaches, such as pressure–volume analysis, which were not performed in the present study.

Therefore, the data underpin a residual effect of prior smoke exposure, but the mechanisms underlying that inference require care. The experimental sequence establishes that smoke exposure preceded infarction and was discontinued during the eight-week recovery period and does not identify the mechanism by which prior exposure influenced later remodeling. Therefore, the present findings support the hypothesis that previous cigarette-smoke exposure may have contributed to the adverse post-infarction phenotype observed in the present study; however, the mechanisms underlying this association remain unresolved. The experimental sequence establishes that cigarette-smoke exposure preceded myocardial infarction and was discontinued during the eight-week recovery period, but it does not identify the biological mechanisms through which previous exposure may have influenced subsequent cardiac remodeling and functional adaptation. Persistent vascular dysfunction, oxidative stress, inflammatory signaling, fibrotic remodeling, altered calcium handling, and autonomic alterations represent biologically plausible pathways that could contribute to the observed phenotype [1,2,30]. In particular, persistent endothelial dysfunction may impair vascular reactivity and contribute to an unfavorable ventricular–vascular interaction, potentially increasing the hemodynamic burden imposed on the post-infarcted myocardium during acute increases in afterload. A priming effect is also conceivable, whereby pre-existing smoke-induced vascular, structural, or cellular alterations may reduce the capacity of the myocardium to adapt to subsequent ischemic injury. Nevertheless, endothelial function, vascular reactivity, inflammatory and oxidative-stress biomarkers, and the molecular or histological pathways underlying these processes were not directly assessed in the present study. Therefore, these mechanisms should be regarded as hypothesis-generating rather than as established causal explanations for the observed cardiovascular phenotype. Future studies incorporating direct assessments of endothelial function, vascular reactivity, inflammatory and oxidative-stress pathways, fibrosis, calcium handling, and autonomic regulation will be necessary to determine whether these mechanisms contribute to the persistent effects of previous cigarette-smoke exposure following myocardial infarction.

The use of female rats is a strength because females remain underrepresented in cardiovascular experimentation; however, it also defines the limits of inference. Female sex and ovarian hormonal status may influence endothelial function, vascular reactivity, myocardial remodeling, and the cardiovascular response to ischemic injury. Estrogen-related signaling, in particular, may modulate endothelial homeostasis and vascular responsiveness, potentially influencing the interaction between cigarette-smoke exposure and post-infarction cardiac remodeling. However, these mechanisms were not directly investigated in the present study. The study did not include male animals, and neither estrous-cycle stage nor circulating ovarian hormones were characterized. Therefore, the present findings describe the integrated cardiovascular phenotype observed in the studied female cohort and should not be interpreted as evidence of a female-specific response. Similarly, the study design does not allow determination of whether hormonal status modified the effects of cigarette-smoke exposure or post-infarction remodeling. Comparative studies including both sexes, with prespecified assessment of estrous-cycle stage, ovarian hormone status, and sex-by-exposure interactions, will be necessary to determine whether the observed responses differ according to sex or reproductive hormonal status.

From a translational perspective, clinical evidence indicating that endothelial dysfunction may contribute to cardiovascular risk even in premenopausal women provides relevant context for considering the interaction between female hormonal status, vascular function, and cardiovascular disease. Nevertheless, such clinical observations should be regarded as contextual rather than directly extrapolated to the present experimental model, particularly because endothelial function and ovarian hormonal status were not directly assessed in the current study [31].

Some additional limitations should be acknowledged. First, the chamber-based exposure model may have introduced cage-level clustering, potentially reducing the independence of individual observations. Second, internal validation of smoke exposure through biomarkers such as cotinine or carboxyhemoglobin was not performed, precluding a direct assessment of individual exposure burden. Furthermore, the use of anesthesia and terminal open-chest instrumentation may have altered cardiovascular loading conditions and hemodynamic responses, thereby potentially influencing the physiological estimates obtained. Finally, the absence of serial hemodynamic assessments limits the ability to characterize temporal adaptations and restricts the generalizability of the findings to conscious, longitudinal physiological conditions.

## 5. Conclusions

Previous cigarette-smoke exposure was associated with a more adverse post-infarction cardiovascular phenotype in female rats, characterized by greater cardiac remodeling and a markedly impaired ventricular functional response to acute phenylephrine-induced afterload stress. Although some cardiovascular alterations were evident under basal conditions, the afterload challenge more clearly revealed the limited capacity of the infarcted heart previously exposed to cigarette smoke to adapt to increased ventricular loading, as evidenced by the pronounced reductions in cardiac output, stroke-volume index, and stroke-work index. Because phenylephrine-induced afterload stress primarily increases systemic vascular resistance and may also influence venous return and preload, the observed changes in +dP/dt, cardiac output, stroke volume, and stroke work should be interpreted as components of an integrated cardiovascular response to increased ventricular loading rather than as direct measures of intrinsic myocardial contractile reserve. Accordingly, the present findings support the presence of impaired ventricular functional reserve under acute afterload stress but do not establish impaired load-independent myocardial contractility.

These findings further suggest that previous cigarette-smoke exposure may contribute to persistent cardiovascular vulnerability after myocardial infarction, even following an eight-week period without continued exposure. However, the mechanisms responsible for this association remain unresolved. Persistent endothelial dysfunction, oxidative stress, inflammatory signaling, fibrotic remodeling, altered calcium handling, and autonomic alterations may represent plausible pathways underlying this phenotype, but these mechanisms were not directly assessed and should therefore be regarded as hypothesis-generating rather than causal explanations.

Importantly, these findings characterize the cardiovascular response of female rats and should not be interpreted as evidence of a female-specific effect, given the absence of a male comparator group and the lack of characterization of estrous-cycle or ovarian hormonal status. Further studies incorporating both sexes, hormonal characterization, direct assessment of endothelial and vascular function, pressure–volume analysis, molecular and histological assessments, and comprehensive evaluation of ventricular–vascular interactions will be important to determine the mechanisms underlying the cardiovascular consequences of previous cigarette-smoke exposure following myocardial infarction.

## Figures and Tables

**Table 1:** Functional and biometric parameters.

Parameters	S	SS	MI	SMI	Effects		
					CS	MI	CS * MI
**Echocardiographic**							
MI size (% of LV)	------	------	45.75 ± 5.47	45.50 ± 7.05	------	------	------
HR (bpm)	242.90 ± 31.97	226.50 ± 33.51	242.40 ± 24.79	240.90 ± 29.00	=0.3681	=0.4888	=0.4548
DA (mm^2^)	28.74 ± 4.03	26.06 ± 1.50	47.10 ± 6.12 *^†^	56.75 ± 4.45 *^†‡^	=0.0196	<0.0001	=0.0003
SA (mm^2^)	14.01 ± 5.26	11.70 ± 0.87	37.26 ± 6.01 *^†^	46.30 ± 4.14 *^†‡^	=0.0301	<0.0001	<0.0008
E wave (s)	79.57± 10.14	76.00 ± 8.46	91.16 ± 13.52	93.41 ± 12.73 *	=0.9457	=0.0004	=0.5083
A wave (s)	33.01 ± 6.21	30.13 ± 9.49	18.74 ± 2.30 *^†^	13.97 ± 2.05 *^†^	=0.0727	<0.0001	=0.4821
E/A	2.54 ± 0.66	2.69 ± 0.77	5.19 ± 0.58 *^†^	7.14 ± 1.05 *^†‡^	=0.0004	<0.0001	=0.0013
FAS (%)	52.43 ± 8.92	54.92 ± 4.64	21.22 ± 4.14 *^†^	17.24 ± 4.73 *^†^	=0.7087	<0.0001	=0.1156
**Cardiac mass**							
AM (mg/g)	0.10 ± 0.01	0.13 ± 0.02	0.31 ± 0.18 *	0.50 ± 0.10 *^†‡^	=0.0047	<0.0001	=0.0316
RVM (mg/g)	0.59 ± 0.08	0.78 ± 0.17	0.87 ± 0.18	1.04 ± 0.77 *	=0.0230	=0.0014	=0.8765
LVM (mg/g)	1.99 ± 0.14	2.15 ± 0.34	2.67 ± 0.22 *^†^	3.15 ± 0.42 *^†‡^	=0.0032	<0.0001	=0.1119
Car (mg/g)	2.68 ± 0.16	3.07 ± 0.37	3.85 ± 0.36 *^†^	4.69 ± 0.59 *^†‡^	<0.0001	<0.0001	=0.0938
**Pulmonary congestion**							
Water contents (%)	78.30 ± 1.70	78.25 ± 1.66	81.00 ± 1.50 *^†^	81.30 ± 1.33 *^†^	=0.1480	<0.0001	=0.3915

Values expressed as mean ± SEM of sham (S), smoker (SS), myocardial infarcted (MI) and smoker + myocardial infarcted (SMI) groups for body mass, myocardial size (MI), LV end-diastolic (DA) and end-systolic (SA) transverse areas, E and A wave velocities and E/A wave ratio, fractional area shortening (FAS), relative atrium mass (AM), relative right ventricular (RVM), relative left ventricular (LVM) mass, relative cardiac mass (Car). CS: cigarette smoke. MI: myocardial infarction. CS * MI: interaction. * vs. S group (*p* < 0.05). † vs. SS group (*p* < 0.05). ‡ vs. MI group (*p* < 0.05).

**Table 2:** Basal hemodynamic parameters.

Parameters	S	SS	MI	SMI	Effects		
					CS	MI	CS * MI
**Right ventricle**							
RVSP (mmHg)	24.00 ± 3.60	21.13 ± 3.87	39.00 ± 5.36 *^†^	38.33 ± 12.45 *^†^	=0.6361	>0.0001	=0.4829
RVDP (mmHg)	0.80 ± 1.22	1.12 ± 1.12	2.55 ± 1.23 *	2.20 ± 1.20 *	=0.8668	=0.0011	=0.4573
+dP/dt (mmHg·s)	3248.00 ± 506.30	2191.00 ± 689.60	1698.00 ± 873.10 *	1472.00 ± 534.50 *	=0.0027	<0.0001	=0.0417
−dP/dt (mmHg·s)	2282.00 ± 365.70	1536.00 ± 517.80 *	1494.00 ± 777.10 *	1202.00 ± 371.40 *	=0.0037	=0.0023	=0.1623
**Left ventricle**							
HR (bpm)	376.60 ± 14.69	355.60 ± 20.88	316.10 ± 16.52	371.70 ± 16.30	=0.5741	=0.1014	=0.0138
LVSP (mmHg)	116.60 ± 7.23	119.10 ± 16.35	107.80 ± 4.49	108.40 ± 18.81	<0.0001	=0.2077	<0.0001
LVEDP (mmHg)	3.30 ± 1.25	4.00 ± 2.50	17.11 ± 5.53 *^†^	18.10 ± 4.84 *^†^	<0.0001	=0.6374	=0.0005
+dP/dt (mmHg·s)	8663.00 ± 767.50	10610.00 ± 1160.00 *	6054.00 ± 1122.10 *^†^	4611.00 ± 1100.50 *^†‡^	=0.6099	<0.0001	<0.0001
−dP/dt (mmHg·s)	6090.00 ± 551.20	5425.00 ± 1160.00	4325.00 ± 561.90 *^†^	3655.00 ± 751.40 *^†^	=0.0090	<0.0001	=0.8553
CO (mL·min^−1^)	50.40 ± 6.97	46.88 ± 4.99	41.00 ± 7.43	38.80 ± 3.11 *	=0.1557	=0.0034	=0.6495
CI (mL·beat^−1^/kg)	206.80 ± 25.32	189.50 ± 25.44	164.10 ± 24.09 *	145.20 ± 42.76 *^†^	=0.0681	=0.0001	=0.9471
SV (mL·beat^−1^)	0.13 ± 0.01	0.13 ± 0.02	0.13 ± 0.02	0.10 ± 0.03	=0.1004	=0.0995	=0.1148
SVI (mL·beat^−1^/kg)	0.56 ± 0.07	0.54 ± 0.10	0.52 ± 0.10	0.39 ± 0.12 *^†‡^	=0.0443	=0.0175	=0.0721
SW (gm·min^−1^)	0.20 ± 0.03	0.20 ± 0.03	0.16 ± 0.03	0.12 ± 0.04 *^†^	=0.0811	<0.0001	=0.1008
SWI (gm·min^−1^/kg)	0.85 ± 0.17	0.84 ± 0.17	0.65 ± 0.14 *	0.46 ± 0.3 *^†^	=0.0718	<0.0001	=0.0699

Values expressed as mean ± SEM of sham (S), smoker (SS), myocardial infarcted (MI) and smoker + myocardial infarcted (SMI) groups of right ventricular systolic pressure (RVSP), right ventricular diastolic pressure (RVDP), left ventricular systolic pressure (LVSP), left ventricular end diastolic pressure (LVEDP), first derivative positive (+dP/dt) and negative (−dP/dt) of left ventricular pressure, cardiac output (CO), cardiac index (CI), stroke volume (SV), stroke volume index (SVI), stroke work (SW), stroke work index (SWI). CS: cigarette smoke. MI: myocardial infarction. CS * MI: interaction. * vs. S group (*p* < 0.05). † vs. SS group (*p* < 0.05). ‡ vs. MI group (*p* < 0.05).

## Data Availability

The raw data supporting the conclusions of this article will be made available by the authors upon reasonable request.

## Abbreviations

The following abbreviations are used in this manuscript:

MI	Myocardial infarction
CS	Cigarette smoke
NIH	Guide for the Care and Use of Laboratory Animals
S	Sham
SS	Smoker
SMI	Smoker with Myocardial Infarction
CAO	Coronary artery occlusion
PHE	Phenylephrine
LV	Left ventricle
RV	Right ventricle
RVSP	Right ventricular systolic pressure
RVDP	Right ventricular diastolic pressure
LVSP	Left ventricular systolic pressure
LVEDP	Left ventricular end-diastolic pressure
HR	Heart rate
+dP/dt	First derivative positive of left ventricular pressure
−dP/dt	First derivative negative of left ventricular pressure
CO	Cardiac output
CI	Cardiac index
SV	Stroke volume
SVI	Stroke volume index
SW	Stroke work
SWI	Stroke work index
DA	LV end-diastolic transverse areas
SA	LV end-systolic transverse areas
FAS	Fractional area shortening
AM	Relative atrial mass
RVM	Relative right ventricular mass
LVM	Relative left ventricular mass
Car	Relative cardiac mass
SEM	Standard error of the mean
